# Cerebral infarction associated with adenomyosis: Two case reports with mechanistic insights and multidisciplinary management

**DOI:** 10.1097/MD.0000000000045294

**Published:** 2025-10-17

**Authors:** Shan Jiang, Liuqing Yang, Jiaqi Wen, Yuanyuan Cao, Wanting Ji, Zanhui Jia

**Affiliations:** a Department of Obstetrics and Gynecology, the Second Hospital of Jilin University, Changchun, Jilin, China.

**Keywords:** abnormal uterine bleeding (AUB), adenomyosis, cerebral infarction (CI), integrated pathogenesis, multidisciplinary treatment

## Abstract

**Rationale:**

Benign gynecological disorders causing abnormal uterine bleeding (AUB), such as adenomyosis, may trigger recurrent thrombosis similar to Trousseau syndrome. Trousseau syndrome is characterized by recurrent thrombotic events in patients with malignant tumors. We report 2 patients with adenomyosis who experienced recurrent cerebral infarction (CI) associated with AUB, exploring the mechanistic insights and highlighting a multidisciplinary approach to treatment.

**Patient concerns:**

One patient suffered 2 episodes of AUB and CI, following repeated failure of conservative therapies. The other patient experienced 3 CI events on the second day of menstruation.

**Diagnoses:**

Both patients were diagnosed with adenomyosis complicated by CI.

**Interventions:**

The first patient underwent conservative treatments, including gonadotropin-releasing hormone agonist, uterine curettage, and levonorgestrel-releasing intrauterine system insertion. The second patient received uterine artery embolization to suppress menstruation, followed by continuous gonadotropin-releasing hormone agonist therapy. Both patients received neuroprotective therapy to stabilize neurological symptoms.

**Outcomes:**

Both patients experienced temporary relief of AUB and neurological symptoms after each episode with conservative treatment. As of now, Patient 1 has experienced multiple episodes of symptom recurrence despite conservative treatment failures and has declined hysterectomy, while Patient 2 has not had any further recurrence.

**Lessons:**

This report highlights the multifactorial pathogenesis of adenomyosis complicated by CI, emphasizing the shared pathway of hypercoagulability, endothelial injury, thrombus formation, and embolic migration. A multidisciplinary, staged management approach is crucial, with acute-phase focus on reperfusion therapy and AUB control, followed by conservative treatments or hysterectomy for long-term management. Neuroprotection and the management of comorbidities are integral throughout. Hysterectomy remains the most effective strategy to prevent recurrence. The proposed framework provides evidence-based guidance for managing these complex cases, with implications for clinical practice and future research.

## 1. Introduction

Malignant tumors induce recurrent thrombosis through mechanisms such as the production of mucinous glycoproteins, tissue factor (TF) release, and coagulation cascade activation, which is known as Trousseau syndrome.^[1]^ Adenomyosis is a common benign gynecological disorder characterized by the abnormal infiltration of endometrial stroma and glands into the myometrium, often clinically presenting with abnormal uterine bleeding (AUB), dysmenorrhea, and infertility.^[2]^ Previous studies have reported that adenomyosis can also lead to Trousseau-like syndrome, with cerebral infarction (CI) being the most common^[3–18]^ (Table 1).

**Table 1:** Patients with adenomyosis complicated with infarction reported in previous literature.

No	Reference	Gynecological diseases	Age/recurrence time (since the last attack)	Hb (g/L, >115)	D-dimer (<0.5 μg/mL)	CA125 (U/mL, < 35)	CA199 (U/mL, < 37)	Cerebral infarction and bleeding time (d)	Gynecological treatment	Site of embolization	Stenosis or occlusion of the carotid or cerebral arteries	Treatment for cerebral infarction				Recurrence	Timing of surgery (from the time of first symptoms)
												Neurotrophic	Anticoagulation	Antiplatelet	Surgery		
**1**	Yin (2018)^[3]^	Adenomyosis	34	134	1.05	937.1	462.5	The 1st day of menstruation	/	Cerebral infarction	No	/	/	/	/	/	/
**2**	Yin (2018)^[3]^	Adenomyosis	37	108	2.34	735.7	43.2	The 2nd day of menstruation	/	Cerebral infarction	No	/	/	/	/	/	/
**3**	Yin (2018)^[3]^	Adenomyosis	46	121	12.04	546.5	1076.6	The 2nd day of menstruation	Hysterectomy	Cerebral infarction	Yes	/	/	/	/	No	5 mo
**4**	Yasuda (2022)^[4]^	Adenomyosis	47	113	3.8	90.3	52.3	The 3rd day of menstruation	/	Cerebral infarction	Yes	Edaravone	Unfractionated Heparin, Edoxaban	/	/	Yes	/
			1st recurrence (after 45 d)	/	4..2	/	/	During menstruation	Hysterectomy	Cerebral infarction, left renal infarction.	/	/	Unfractionated heparin	/	/	No	About 50–60 d
**5**	Aso (2018)^[5]^	Adenomyosis	44	103	17	2115	1824	During menstruation	/	Cerebral infarction, Splenic infarction	Yes	Edaravone	Heparin, Rivaroxaban	/	/	Yes	/
			1st recurrence (after 31 d)	Anemia was aggravated after anticoagulant therapy.	/	561	/	The 2nd day of menstruation	GnRH agonist	New Cerebral infarction	/	/	Rivaroxaban, Warfarin	/	/	Yes	About 6 mo
			2nd recurrence (after 5 mo)	/	/	1291.6	/	Irregular menstrual bleeding had continued for a month.	Hysterectomy	New Cerebral infarction	/	/	Warfarin	/	/	No	/
**6**	Kimura (2012)^[6]^	Adenomyosis	45	84	1.1	159	/	History of heavy menstrual bleeding, not in the menstrual phase	GnRH agonist	Cerebral infarction, left fingers infarcts, Thrombi in the brachiocephalic trunk, and left subclavian artery.	No	/	Heparin	Antiplatelet therapy	/	/	/
**7**	Kimura (2012)^[6]^	Adenomyosis	44	70	/	/	/	/	GnRH agonist	Cerebral infarction, Splenic infarction	No	/	Heparin, Warfarin		/	/	/
**8**	Kimura (2012)^[6]^	Adenomyosis	50	69	0.57	42.6	/	During menstruation	GnRH agonist	Cerebral infarction	No	/	/	Aspirin	/	/	/
**9**	Kimura (2012)^[6]^	Adenomyosis	42	86	6	1750	/	During menstruation	GnRH agonist	Cerebral infarction	/	/	/	Anti-platelets treatment	/	Yes	/
	Yamashiro (2012)^[7]^		1st recurrence (After 1 yr)	/	4.1	907	/	1 wk after menstruation	GnRH agonist	New Cerebral infarction	No	/	Heparin, Warfarin	/	/	/	/
**10**	Okazaki (2018)^[8]^	Adenomyosis	42	/	1.4	395	/	Unknown	/	Cerebral infarction	Yes	/	Warfarin	/	/	No	/
**11**	Okazaki (2018)^[8]^	Adenomyosis	50	/	3.7	143	/	/	/	Cerebral infarction	Yes	/	Rivaroxaban	/	/	No	/
**12**	Arai (2022)^[9]^	Adenomyosis	50	92	6.4	999	112	The 2nd day of menstruation	GnRH agonist	Cerebral infarction	No	/	Heparin, Apixaban	/	/	Yes	14 d
			1st recurrence (after 7 d)	/	/	/	/	/	Hysterectomy	New Cerebral infarction	/	/	Apixaban	/	/	No	
**13**	Zhao (2020)^[10]^	Adenomyosis	34	112	27.4	937	/	During menstruation	A history of GnRH agonist therapy was discontinued for 6 mo.	Cerebral infarction	Yes	/	Heparin	Clopidogrel	/	No	/
**14**	Kim (2018)^[11]^	Adenomyosis	49	99	6.99	379	69.2	10 d after menstruation	Hysterectomy	Cerebral infarction, NBTE	No	/	Heparin, Vitamin K antagonist	/	/	No	14 d
**15**	Aiura (2021)^[12]^	Adenomyosis, multiple uterine leiomyoma	48	82	79.3	/	/	Heavy uterine bleeding	/	New Splenic Infarction	/	/	/	/	/	/	/
			Progress 5 d later	/	/	3536	892	Heavy uterine bleeding	Hysterectomy	Cerebral infarction	Yes	/	/	/	Endovascular thrombectomy	Yes	11 d
			Recurrence of other sites after 9 d	/	/	/	/	/	/	Pulmonary thromboembolism	/	/	Heparin, Edoxaban	/	/	No	/
**16**	Zhang (2022)^[13]^	Adenomyosis	38	61	/	/	/	Abnormal uterine bleeding for 1 mo	Hysterectomy	Moyamoya disease history, Cerebral infarction	Yes	Edaravone, Ganglioside	Argatroban	Aspirin	Superficial temporal artery-middle cerebral artery bypass combined with encephalo-duro-myo-synangiosis muscle adhesion.	/	14 d
**17**	Hijikata (2016)^[14]^	Adenomyosis	59	/	7	334.8	/	/	Stop hormone replacement therapy.	Cerebral infarction, NBTE	/	/	Heparin	/	/	No	/
**18**	Uchino (2018)^[15]^	Adenomyosis	48	85	1.9	901	1791	Abnormal uterine bleeding for 1 mo	Hysterectomy	Cerebral infarction, NBTE	Yes	/	Heparin, Warfarin	/	/	No	About 3 mo
**19**	Morishima (2023)^[16]^	Adenomyosis	42	132	<0.5	293	<2	The 1st day of menstruation	/	Cerebral infarction	/	Edaravone	Heparin, Edoxaban	/	/	Yes	/
			9 d after hospital discharge	/		743		The 2nd day of menstruation	GnRH agonist, Hysterectomy	New Cerebral infarction	/	Edaravone	Heparin, Edoxaban	/	/	No	111 d
**20**	Nishioka (2014)^[17]^	Adenomyosis	47	76	6.3	784.6	/	/	Hysterectomy	Cerebral dural venous thrombosis	Yes	/	/	Antiplatelet treatment	/	No	1 yr
**21**	Tadokoro (2013)^[18]^	Adenomyosis	57	/	11.6	1470	/	/	/	Cerebral infarction, PFO, Paradoxical cardioembolism	/	/	/	/	/	/	/

Abbreviation: GnRH = gonadotropin-releasing hormone.

This article describes 2 cases of AUB caused by adenomyosis, complicated by CI. In one case, CI occurred after anemia secondary to bleeding, but after 2 rounds of conservative gynecological treatment, including uterine curettage, gonadotropin-releasing hormone agonist (GnRH-α), and the levonorgestrel intrauterine system (LNG-IUS) insertion, long-term therapeutic efficacy was not achieved, with bleeding and CI recurring. Another patient experienced recurrent CI linked to the menstrual cycle, but no further episodes occurred after uterine artery embolization (UAE) and GnRH-α induced successful amenorrhea.

Through case analyses and a literature review, this article systematically integrates the multifactorial mechanisms of CI associated with adenomyosis, explores key interventions for the primary gynecological disease, and discusses strategies for preventing and managing neurological complications. It also establishes a phased, multidisciplinary collaborative treatment pathway, providing new clinical insights and a practical framework for the recognition and management of such cases.

## 2. Case report

### 2.1. Patient 1

**Figure 1.**
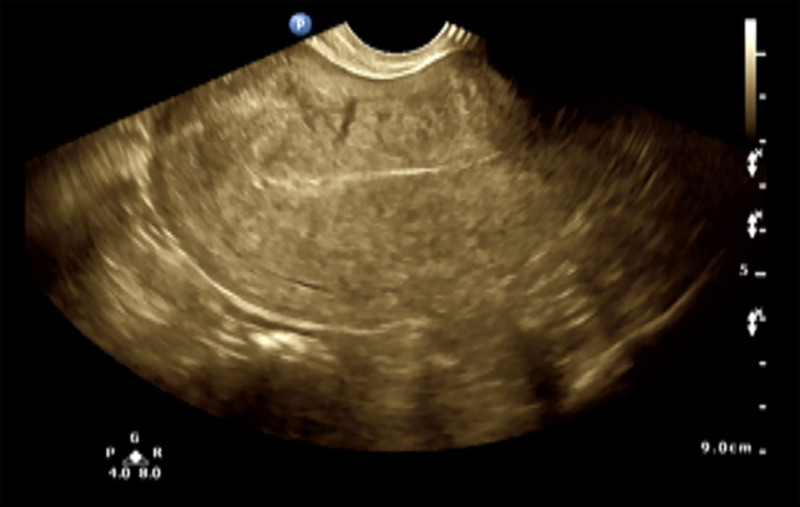
Gynecological ultrasound image of Patient 1.

**Figure 2.**
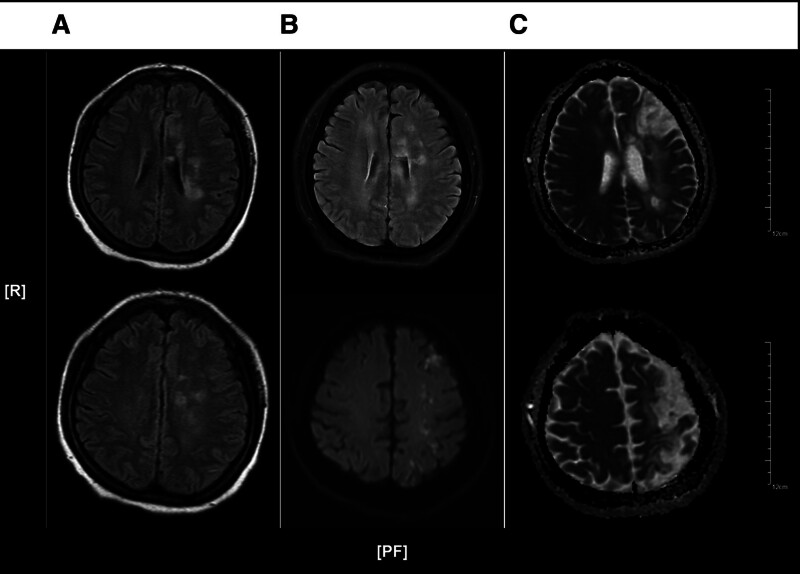
Representative brain magnetic resonance imaging (MRI) scans from Patient 1 during 3 separate admissions, demonstrating progressive cerebral infarction characterized by newly developed ischemic lesions at each hospitalization: (A) first admission, (B) second admission, and (C) third admission.

A 47-year-old female was admitted due to continuous AUB for 20 days, followed by 3 days of sudden right-sided motor impairment. She had a 5-year history of hypertension, newly diagnosed diabetes with poor control, and denied any abnormal menstrual history, oral contraceptive use, coronary artery disease, smoking history, or family history. Laboratory tests revealed the hemoglobin of 67 g/L (normal 115–150 g/L), Cancer Antigen 125 (CA125) of 17.30 U/mL (normal < 35 U/mL), CA199 of 7.0 U/mL (normal < 37 U/mL), and D-dimer of 0.29 µg/mL (normal 0–1.00 µg/mL). Dyslipidemia [hypertriglyceridemia and low high-density lipoprotein cholesterol (HDL-C)] was noted, while homocysteine, coagulation function, platelet count, myocardial enzymes, myocardial markers, and B-type natriuretic peptide (BNP) were normal. Gynecological ultrasound showed adenomyosis (Fig. 1). Head magnetic resonance imaging (MRI) showed acute or subacute infarcts in the left basal ganglia, corona radiata, centrum semiovale, and left side of the corpus callosum (Fig. 2A). Carotid ultrasound showed intimal thickening and plaques, with 50% to 69% stenosis at the right internal carotid and vertebral arteries. Intracranial vascular ultrasound (TCCS) and bilateral lower limb deep vein ultrasounds showed no abnormalities. Cardiac ultrasound showed concentric left ventricular hypertrophy and mitral regurgitation, and the electrocardiogram (ECG) showed left ventricular hypertrophy and abnormal ST-T changes.

Treatment included anemia correction, blood pressure management, glucose control, oral atorvastatin, and the use of Xingnaojing and calf blood deproteinized for neuroprotection. Given the acute phase of CI and the patient’s preference for uterus preservation, a uterine curettage and GnRH-α were administered, achieving a 29-month symptom remission.

**Figure 3.**
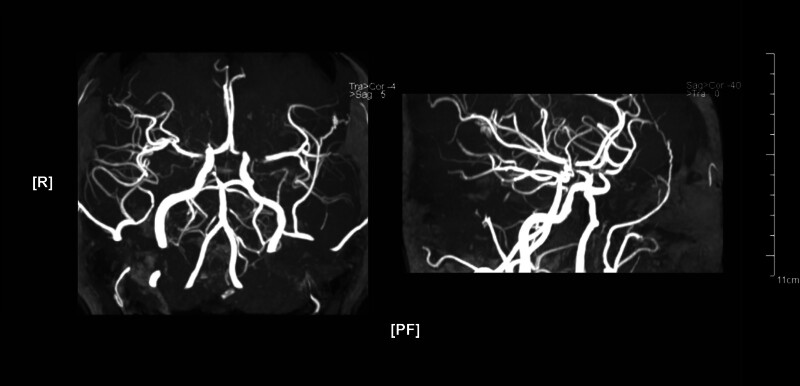
Magnetic resonance angiography (MRA) from Patient 1 during the second admission, showing multiple stenoses of the carotid and cerebral arteries.

After that, she was readmitted for persistent AUB with dizziness and fatigue. Hemoglobin decreased to 51 g/L, and dyslipidemia persisted. CA125, CA199, D-dimer, homocysteine, antineutrophil cytoplasmic antibody (ANCA)-associated vasculitis test, antinuclear antibody (ANA) panel, immunoglobulins, complement C3, C4, rheumatoid factor, anti-streptolysin O (ASO) antibodies, coagulation function, platelet count, myocardial enzymes, myocardial markers, and BNP were all normal. Abdominal and chest computed tomography (CT) scans did not reveal any malignant lesions. Cardiac ultrasound, ECG, and bilateral lower limb deep vein ultrasounds showed no abnormalities. AUB improved after 1 week of uterine contraction and anemia correction, but the patient suddenly developed complete motor aphasia, right-sided central facial and tongue paralysis. Repeat brain MRI showed new infarcts (Fig. 2B). Magnetic resonance angiography (MRA) and carotid ultrasound indicated cerebral and carotid atherosclerosis, with stenosis in the P1 segment of the right posterior cerebral artery, bilateral middle cerebral arteries, bilateral anterior cerebral arteries, the left distal internal carotid artery, the right internal carotid artery at its origin, and the opening of the right vertebral artery (Fig. 3). Given the patient’s anemia and multiple comorbidities, antithrombotic and vascular interventions were not pursued. A neuroprotective regimen (edaravone, butylphthalide, and calf blood deproteinized) combined with Chinese medicine preparations (Xingnaojing injection and ginkgo biloba) was initiated, resulting in significant neurological improvement. Subsequently, uterine curettage and LNG-IUS placement were performed.

Six months postoperatively, the patient experienced LNG-IUS expulsion and presented with recurrent AUB. Seven months later, she was readmitted with AUB and right lower limb weakness. Hemoglobin dropped to 73 g/L, erythropoietin was 109.44 mIU/mL (normal 2.59–18.5 mIU/mL), and dyslipidemia persisted. Brain MRI showed new CI in the right frontal-parietal lobe (Fig. 2C). Homocysteine, coagulation function, platelet count, myocardial enzymes, cardiac markers, BNP, and ECG were normal. Neuroprotective treatment was continued, and the patient improved after 4 days of therapy. Despite recommendations for a hysterectomy to prevent recurrence of CI, the patient declined the procedure at her last follow-up.

### 2.2. Patient 2

**Figure 4.**
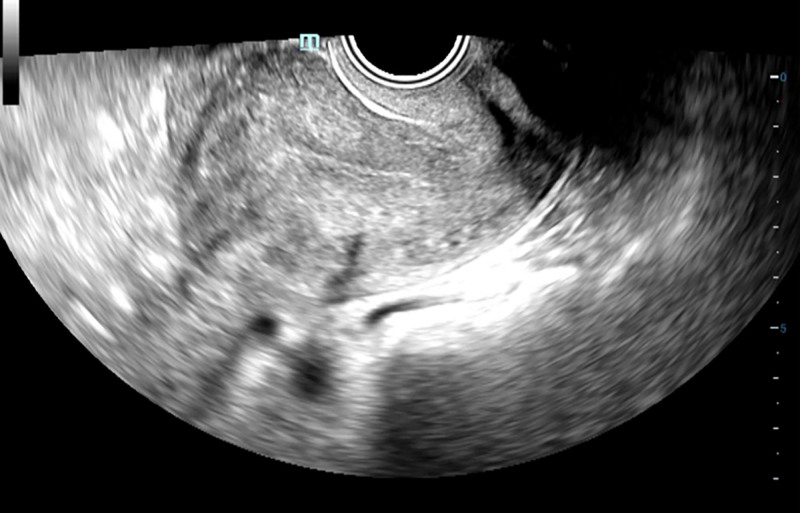
Gynecological ultrasound image of Patient 2.

A 48-year-old woman with a 5-year history of adenomyosis (Fig. 4) presented with progressively worsening dysmenorrhea and menorrhagia, without standardized treatment. Five months, 2 months, and 1 month prior to admission, on the second day of each menstrual cycle, she experienced acute neurological deficits, including right-hand tremors, right-sided motor impairment, and slurred speech, all of which were diagnosed as acute CI. MRI confirmed multiple acute cerebral infarcts in the left frontal lobe, parietal lobe, centrum semiovale, and basal ganglia region. Local stenosis was observed in the M1 segment of the left middle cerebral artery and the C6–7 segment of the left internal carotid artery. TCCS revealed severe stenosis or occlusion of the left internal carotid artery beyond the ophthalmic artery, compensatory stenosis in the left posterior cerebral artery, and mild stenosis in the right posterior cerebral artery. Carotid ultrasound showed a solitary plaque formation in the right carotid artery. The patient also exhibited dyslipidemia (hypertriglyceridemia and low HDL-C) but normal homocysteine. After each stroke, she received blood transfusions and neuroprotective drugs, including edaravone and butylphthalide. She was discharged in stable condition with residual mild right-sided motor impairment and slurred speech, continuing atorvastatin and clopidogrel.

**Figure 5.**
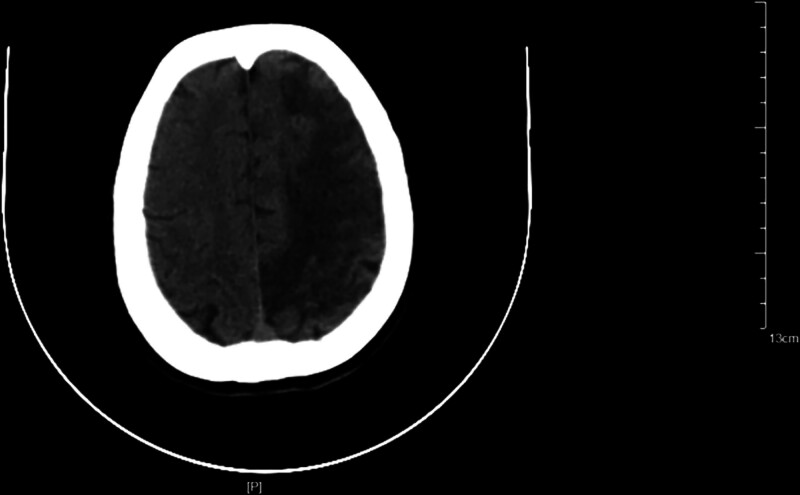
Brain computed tomography (CT) scan from Patient 2 at admission, showing a left frontal-parietal infarction and multiple bilateral lacunar infarctions.

Twenty-seven days after her last menstrual period, she was referred to the gynecology department for further treatment. The patient had hypertension, diabetes with poor control, and both parents had a history of CI. Laboratory examination showed elevated levels of CA125 (254.80 U/mL) and CA199 (83.17 U/mL) along with a platelet hyperaggregation state [aggregation rates>85% in response to adenosine diphosphate (ADP), adrenaline, collagen, and arachidonic acid (AA)]. Head CT indicated a left frontal-parietal infarction and multiple bilateral lacunar infarctions (Fig. 5). Hemoglobin levels, D-dimer, homocysteine, coagulation function, platelet count, myocardial enzymes, cardiac markers, BNP, ECG, and cardiac ultrasound were normal.

To manage the patient’s impending menstruation and prevent antithrombotic therapy from exacerbating menstrual bleeding, she underwent uterine and internal iliac artery embolization on the third hospital day. On the fourth postoperative day, GnRH-α was injected, and aspirin was added to the treatment regimen.

Twenty-four days later, breakthrough menstruation occurred due to the “flare-up effect” of GnRH-α. On the fifth day of menstruation, she was readmitted to the neurology department for a new CI, which improved after neuroprotective therapy. GnRH-α was administered once every 28 days, successfully maintaining amenorrhea to date, with no recurrence of AUB or CI. The patient declined vascular intervention and hysterectomy.

## 3. Discussion

### 3.1. Pathogenesis

Both patients with adenomyosis in this study received conservative treatment from the gynecology and neurology departments for AUB complicated by CI, and neither underwent surgical intervention. Both exhibited comorbidities, including hypertension, poorly controlled diabetes and dyslipidemia, along with varying degrees of cervical or cerebral vascular stenosis and atherosclerosis (Table 2).

**Table 2:** Clinical characteristics, treatment, and outcomes of patients in this report.

No.	Gynecological diseases	Age/recurrence time (since the last attack)	Hb (g/l, >115)	D-dimer (<0.5 μg/mL)	CA125 (U/mL, < 35)	CA199 (U/mL, < 37)	CI and bleeding time (d)	Gynecological treatment	Site of embolization	Stenosis or occlusion of the carotid or cerebral arteries	Treatment For CI				Recurrence	Timing of surgery (from the time of first symptoms)
											Neurotrophic	Anticoagulation	Antiplatelet	Surgery		
1	Adenomyosis	47	67	0.29	17.3	7	Heavy bleeding for 20 d	Curettage, GnRH agonist	Cerebral infarction	Yes	Xingnaojing, calf blood deproteinized	/	/	/	Yes	/
		1st recurrence (after 29 mo)	51	0.44	13	3.38	Heavy bleeding for 30 d	Hysteroscopy, curettage, levonorgestrel intrauterine sustained-release system placement	New cerebral infarction	Yes	Edaravone, Butylphthalide, Xingnaojing, Ginkgo biloba, calf blood deproteinized	/	/	/	Yes	/
		2nd recurrence (after 13 mo)	73	/	/	/	Bleeding	Hemostatic agents	New cerebral infarction	/	Ginkgo biloba, calf blood deproteinized	/	/	/	/	/
2	Adenomyosis	48	/	/	/	/	The 2nd day of menstruation	/	Cerebral infarction	Yes	Edaravone, butylphthalide	/	Clopidogrel	/	Yes	/
		1st recurrence (after 1 mo)	/	/	/	/	The 2nd day of menstruation	/	New cerebral infarction	Yes	Yes	/	/	/	Yes	/
		2nd recurrence (after 2 mo)	/	/	/	/	The 2nd day of menstruation	/	New cerebral infarction	Yes	Yes	/	/	/	Yes	/
		Admitted 25 d later	125	0.5	254.8	83.17	/	Uterine artery and iliac artery embolization, GnRH agonist	/	/	/	/	Clopidogrel, aspirin	/	Yes	/
		28 d after the previous admission	/	/	/	/	The 5th day of menstruation	/	New cerebral infarction	/	Yes	/	/	/	No	/

Abbreviation: GnRH = gonadotropin-releasing hormone.

Notably, although Patient 2 received antiplatelet therapy after the first CI, it did not prevent the recurrence of CI during menstruation. This pattern suggests that the pathogenesis of these patients may extend beyond the traditional large-artery atherosclerotic CI, involving more complex multifactorial interactions.

#### 3.1.1. Mucinous tumor markers: menstruation-linked hypercoagulation, cardioembolism, and atherosclerosis

In patients with malignant tumors and CI, mucinous tumor markers (such as CA125 and CA199) can induce hypercoagulation through bidirectional signaling between neutrophils and platelets, promoting tumor thrombus formation.^[19]^ This is often accompanied by elevated D-dimer levels.^[2]^ These markers are also commonly elevated in patients with adenomyosis, as observed in Patient 2 of this study and in previous reports. Notably, CA125 levels peak at the beginning of menstruation,^[20]^ coinciding with the onset of CI in approximately half of the cases reported in the literature, as well as in Patient 2. Following treatment, CA125 levels typically decrease, and D-dimer levels normalize subsequently.

The shedding of platelet or fibrin thrombi from the heart valves or endocardial surface in patients with nonbacterial thrombotic endocarditis (NBTE) is a major cause of cardioembolism.^[15]^ In addition to malignancy, autoimmune diseases, and atrial fibrillation, abnormally elevated levels of mucinous tumor markers can also increase the risk of NBTE. If a patent foramen ovale (PFO) is present, the risk of ectopic emboli leading to paradoxical embolism at multiple sites throughout the body increases, which is also a common cause of cryptogenic stroke in young patients.^[21]^ Due to the limited detection capability of conventional imaging for small valvular vegetations, most NBTE cases are diagnosed at autopsy.^[3]^ Transesophageal echocardiography (TEE) is more accurate than transthoracic echocardiography (TTE) in identifying these conditions.^[22]^ Therefore, although the TEE, ECG, cardiac markers, and other tests of the 2 patients revealed no evidence of cardioembolism, atrial fibrillation, or PFO, the possibility of cardioembolism in our patients cannot be entirely excluded. Additionally, the left atrial enlargement in Patient 1 has been shown to increase the incidence of cardioembolism through mechanisms such as blood stasis and increased atrial fibrillation risk.^[23]^

Both patients exhibited stenosis and atherosclerosis of the cerebral and carotid arteries. Traditional vascular risk factors (including hypertension, diabetes, dyslipidemia, hyperhomocysteinemia, etc.),^[24]^ and CA125-mediated high blood viscosity may synergistically accelerate the process of atherosclerosis.^[25]^ Further clarification through basic and clinical research is needed.

The threshold at which CA125 can cause CI in patients with adenomyosis requires further investigation, and its clinical application value as a predictive marker deserves deeper exploration.^[9]^

#### 3.1.2. Ectopic endometrium and TF: inflammation and coagulation cascade, accompanied by microthrombosis and fibrinolysis activation

Similar to endometriosis,^[26]^ the local inflammatory response in the ectopic endometrium within the myometrium of adenomyosis patients leads to upregulation of TF expression.^[27]^ As a key initiator of the extrinsic coagulation pathway, TF activates the coagulation cascade, causing hypercoagulation. Studies have also linked TF to menorrhagia and dysmenorrhea.^[28]^

Furthermore, the periodic necrosis of endometrial tissue in the myometrium of patients with adenomyosis may trigger local inflammatory reactions or microbleeding, leading to the formation of fibrin and microthrombi during menstruation. Soluble fibrin not only increases blood viscosity but also promotes its deposition on the surface of vascular endothelial cells, thereby raising the risk of microcirculatory dysfunction. Microthrombi may enter the systemic circulation through the abnormally dilated blood vessels within adenomyosis lesions, serving as a potential source of cerebral embolism. These microthrombi may activate the fibrinolytic system, dissolving the fibrin needed for hemostasis on the uterine cavity surface during menstruation, thus contributing to menorrhagia.^[29]^

#### 3.1.3. Menorrhagia-induced anemia: hypoxia and hemodynamic changes promoting stroke

The menorrhagia caused by various factors mentioned above leads to anemia in patients with adenomyosis, and anemia impacts the cerebral vascular system by altering oxygen delivery, blood viscosity, and blood flow. Anemia-induced hypoxia and inflammation can induce endothelial dysfunction and accelerate atherosclerosis development. Secondary cerebral hypoxia can also lead to neurological damage. At the same time, changes in shear stress and blood viscosity due to anemia can affect platelet aggregation and clot formation, increasing the risk of thrombotic events.^[30]^ The hyperdynamic state caused by anemia may further damage the vascular endothelium, promoting thrombus formation and ectopy.^[31]^ Among the cases in Table 1, 61.9% (13/21) of the patients had varying degrees of anemia, consistent with the symptoms observed in Patient 1.

#### 3.1.4. Infection and immune dysregulation: inflammation amplifying stroke risk

Literature has reported that fever and infection are potential factors for CI in patients with adenomyosis.^[10,32]^ Autoimmune diseases also significantly increase the risk of CI through mechanisms like endothelial inflammation.^[32]^ This also highlights the need to strengthen the differential diagnosis of these potential triggers.

**Figure 6.**
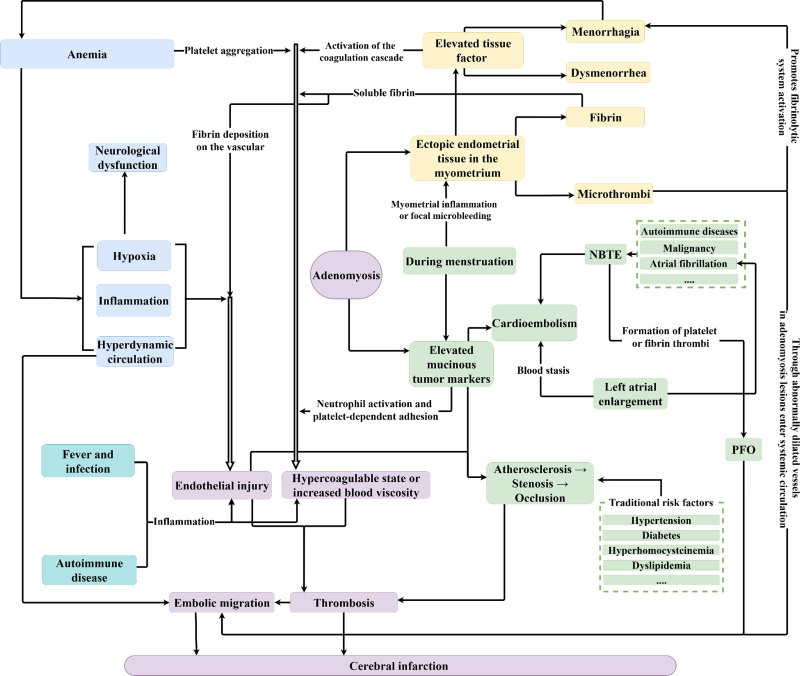
Schematic illustration of the multifactorial mechanisms linking adenomyosis with cerebral infarction. Contributing factors include elevated mucinous tumor markers, ectopic endometrial tissue–induced inflammation, menorrhagia-related anemia, and traditional vascular risk factors. These converge on a shared pathogenic cascade of “hypercoagulable state → endothelial injury → thrombosis → embolic migration”, ultimately leading to cerebral infarction.

In summary, the multiple possible mechanisms of AUB associated with CI in adenomyosis patients ultimately trigger CI through a common pathway of “hypercoagulable state-endothelial injury-thrombosis-embolic malposition” (Fig. 6). A multidimensional assessment system, including hematology, immunology, cardioembolism, and vascular imaging, should be implemented to better elucidate the etiology and inform the development of tailored, precise treatment strategies.

### 3.2. Treatment strategies

**Figure 7.**
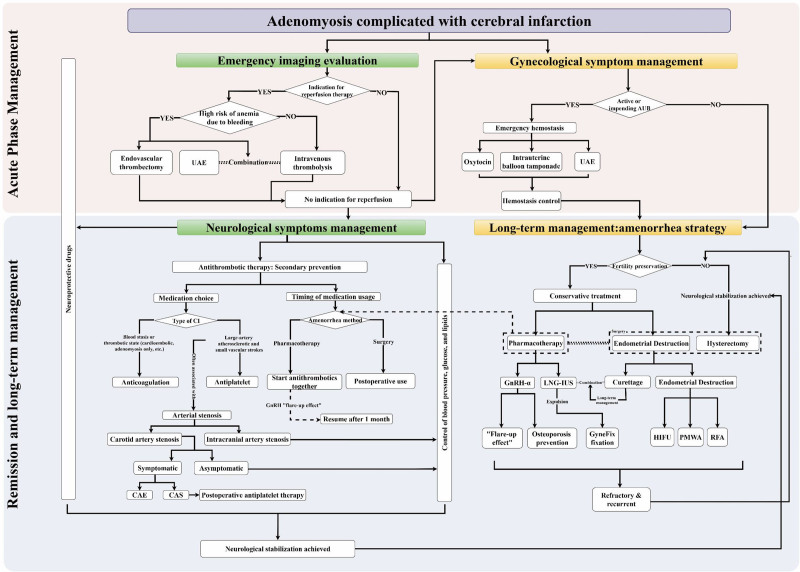
Multidisciplinary, staged management pathway for adenomyosis complicated by cerebral infarction. Acute-phase strategies focus on emergency imaging evaluation, reperfusion decision-making, hemostasis, and neuroprotection. Remission and long-term management emphasize amenorrhea induction (pharmacotherapy, LNG-IUS, endometrial destruction, or hysterectomy), individualized antithrombotic therapy, and control of vascular risk factors. The framework integrates gynecology, neurology, neurosurgery, and interventional radiology to balance hemostasis with thrombosis prevention. AUB = abnormal uterine bleeding, CAS = carotid artery stenting, CEA = carotid endarterectomy, CI = cerebral infarction, GnRH-α = gonadotropin-releasing hormone agonist, HIFU = high-intensity focused ultrasound, LNG-IUS = levonorgestrel-releasing intrauterine system, PMWA = percutaneous microwave ablation, RFA = radiofrequency ablation, UAE = uterine artery embolization.

The treatment goal for these patients is to stabilize neurological function, control the progression of CI, and prevent recurrence, while effectively managing the AUB caused by adenomyosis. Due to the multifactorial nature of this disease and the treatment conflict (i.e., the balance between antithrombotic therapy and hemostasis), collaborative multidisciplinary decision-making is crucial (Fig. 7).

#### 3.2.1. Acute-phase management

##### 3.2.1.1. Imaging assessment and reperfusion decision

In the acute phase, emergency cranial and vascular imaging should be conducted to determine whether intravenous thrombolysis or endovascular thrombectomy with reperfusion therapy is indicated. Although active bleeding is not an absolute contraindication to thrombolysis, the risk of anemia should still be thoroughly assessed.^[33]^ UAE can be performed simultaneously using materials resistant to thrombolytics to achieve amenorrhea.^[34]^ Endovascular thrombectomy is preferable for patients with a high bleeding risk.

##### 3.2.1.2. Neuroprotective therapy

Given the time-sensitive nature of reperfusion therapy, along with technological limitations and the risk of reperfusion injury, only a minority of patients benefit from reperfusion therapy. Therefore, neuroprotective and neurorepair drugs should be initiated in the acute phase. Multiple mechanisms of neuronal death, including intrinsic and extrinsic apoptosis, necroptosis, and inflammatory cell death, contribute to the pathogenesis of CI.^[35]^ Various neurotrophic drug targets have been developed to address these processes, and natural extracts of Chinese herbal medicines have also been gradually applied clinically in recent years.^[36]^ The medications used by the patients in this report and their mechanisms of action are shown in Table 3.^[37–40]^ Further research is needed to identify the most effective drug class for improving neurofunctional outcomes and to explore their potential impact on the hypercoagulable state and AUB in adenomyosis. These drugs can help stabilize neurological symptoms early in the course of CI and may be continued to provide time for additional interventions.

**Table 3:** Neuroprotective medications administered in this report and their primary mechanisms of action.

Drug name	Main mechanism of action and additional notes
Butylphthalide^[37]^	Protects mitochondrial function, improves neuronal energy metabolism and functional recovery, and enhances microcirculation.
Edaravone^[37]^	Inhibits oxidative stress response, regulates arachidonic acid metabolism, suppresses cell apoptosis, and eliminates free radicals.
Calf blood deproteinized^[38]^	Enhances tissue oxygenation, reduces inflammatory and apoptotic responses, and promotes ischemic tissue repair.
Xingnaojing Injection^[39]^	Alleviates inflammation, improves antioxidant function, and is the only approved Chinese herbal injection for acute stroke.
Ginkgo biloba^[40]^	Increases blood flow to ischemic regions, prevents membrane damage caused by free radicals, and improves cognitive and behavioral functions.

#### 3.2.2. Secondary stroke prevention and antithrombotic strategies

Antithrombotic therapy is the cornerstone of secondary prevention in CI, mainly including anticoagulant therapy and antiplatelet strategies. Antiplatelet drugs are often preferred for large-artery atherosclerotic and small vascular strokes, such as aspirin and clopidogrel. For strokes caused by blood stasis or a thrombophilic state, such as cardiogenic embolism, anticoagulation therapy is selected due to the tendency to form red thrombi, with options including heparin, warfarin, and rivaroxaban.^[41]^ Adenomyosis patients are also prone to hypercoagulability, and previous reports have used anticoagulant drugs, among which heparin is the most commonly used.

Alongside antithrombotic therapy, controlling traditional stroke risk factors such as hypertension, diabetes, and dyslipidemia can reduce the risk of recurrence and delay the progression of atherosclerosis. Carotid endarterectomy (CEA) and carotid artery stenting can benefit patients with symptomatic carotid artery stenosis. However, asymptomatic patients are best treated with intensive medications (such as statins), which is also recommended for intracranial artery stenosis.^[42]^

#### 3.2.3. Timing of hemorrhage control and antithrombotic therapy

Antithrombotic therapy may induce or exacerbate AUB in adenomyosis patients, thereby worsening CI.^[43]^ Unlike the conventional initial antithrombotic treatment for CI, such patients should give priority to using oxytocin, intrauterine balloon tamponade,^[44]^ UAE, or other measures to control active or impending AUB. Amenorrhea treatment should be initiated, and the timing of antithrombotic therapy should be determined based on the amenorrhea method and the type of CI: pharmacological amenorrhea can be synchronized with antithrombosis, while for surgical amenorrhea, antithrombotic therapy should be paused perioperatively and resumed once the postoperative risk decreases.

#### 3.2.4. Long-term management and curative treatment

For women desiring fertility preservation, conservative approaches should be prioritized, including pharmacotherapy (GnRH-α and LNG-IUS) and endometrial destruction (curettage and endometrial ablation).

##### 3.2.4.1. Conservative treatment – pharmacotherapy

GnRH-α initially activates the pituitary-gonadal axis, causing a transient increase in estrogen levels (“flare-up effect”), which may trigger breakthrough bleeding. Continued medication cause downregulation of GnRH-α receptors and desensitization, thereby inhibiting gonadotropin secretion and inducing pharmacological menopause, which helps control abnormal endometrial hyperplasia and bleeding.^[45]^ During treatment, calcium and vitamin D must be supplemented in advance, and bone status should be monitored to avoid irreversible osteoporosis.^[46]^ Antithrombotic drugs can be selectively resumed 1 month after stabilization of pharmacological menopause.

The LNG-IUS offers a reversible option, delivering local progesterone to reduce adenomyotic lesions. However, in patients with an enlarged uterine cavity, LNG-IUS expulsion is common, leading to rapid progesterone withdrawal and potential breakthrough bleeding. Therefore, the preventive fixation with a GyneFix device represents a significant improvement for the stabilization of the LNG-IUS in such cases.^[47]^

In addition, hormone replacement therapy, commonly used for conventional adenomyosis patients, is contraindicated in those with CI, because long-term hormone replacement therapy may elevate CA125 levels and may increase the risk of CI.^[14]^

##### 3.2.4.2. Conservative treatment – endometrial destruction

To minimize drug-related side effects, patients with endometrial thickening can also choose uterine curettage. However, this procedure offers only temporary relief and is not a long-term solution, making a combination with LNG-IUS preferable for sustained endometrial management.

Recently, endometrial ablation techniques – such as high-intensity focused ultrasound, percutaneous microwave ablation (PMWA), and radiofrequency ablation (RFA) – have been gradually applied to the conservative treatment of adenomyosis. Among these, RFA demonstrates the greatest efficacy in reducing recurrence rates^[48]^ and can achieve an effect similar to uterine amenorrhea (“Asherman syndrome”).^[49]^

These endometrial-destructive procedures are generally brief, minimizing the risk of prolonged anesthesia-related cerebral hypoperfusion and potential exacerbation of CI.

##### 3.2.4.3. Surgical treatment and timing of surgery selection

When conservative treatments fail, after the first recurrence of AUB with CI symptoms, or in a patient without fertility preservation desires, a timely hysterectomy after achieving hemostasis, amenorrhea, and stable neurologic symptoms remains the most effective intervention to prevent recurrence.

Literature suggests that for patients with only elevated CA125, surgery should be performed within 7 days after the first symptom onset, following neurological stabilization.^[9]^ Although elective noncardiac surgery within 3 months of CI is generally associated with higher cardiovascular risk, including stroke, timely hysterectomy in this patient population represents the most definitive strategy to prevent secondary CI. Therefore, surgical timing should be optimized to balance neurological stability with effective management of adenomyosis.^[50]^

### 3.3. Cases analysis

In Patient 1, the pathogenesis likely involves local effects of ectopic endometrial tissue in the myometrium, upregulation of TF, posthemorrhagic anemia, and arterial atherosclerosis. The first episode was managed with curettage combined with GnRH-α, achieving 29 months of remission before the first recurrence. The second recurrence occurred 6 months after curettage and LNG-IUS insertion, due to the expulsion of the device. Given the limited durability of previous treatments, hysterectomy may represent a more definitive option, along with long-term control of blood pressure, blood glucose, and lipid levels. Antiplatelet therapy should be initiated once bleeding is stabilized.

In Patient 2, pathogenesis may involve local effects of ectopic endometrium in the myometrium, TF upregulation, elevated CA125 levels, and their fluctuation during menstruation, posthemorrhagic anemia, and atherosclerosis. Initial management included UAE to suppress menstruation, followed by continuous GnRH-a therapy. Although a flare-up effect caused breakthrough bleeding and new-onset stroke, no further recurrence occurred after reaching a stable drug-induced menopause, with normal bone mineral density. The patient maintained stable antiplatelet therapy and achieved good outcomes with controlled blood pressure, glucose, and lipid levels. Future treatment may include endovascular intervention or hysterectomy, depending on disease progression and patient preference.

Both patients were distressed by recurrent AUB and CI but felt relieved and more confident after multidisciplinary, individualized management.

## 4. Conclusion

Although adenomyosis is a common benign gynecological disease, some patients may develop new CI secondary to AUB, which requires high clinical attention.

The pathogenesis is multifactorial, involving elevated mucinous tumor markers, upregulation of TF in ectopic endometrial tissue in the myometrium, menstrual anemia, and vascular lesions. These factors interact and culminate in CI through the shared pathway of “hypercoagulable state – endothelial injury – thrombus formation – embolus migration.”

In terms of treatment, this study has established a multidisciplinary collaborative diagnostic and treatment pathway encompassing the acute phase, remission and long-term management. Key departments include gynecology, neurology, neurosurgery, interventional radiology, and internal medicine, to achieve a dynamic balance between hemostasis and thrombosis prevention.

During the acute phase, after imaging assessment, neurology leads the initiation and maintenance of neuroprotective and nutritional treatments. Depending on the bleeding risk, intravenous thrombolysis and surgical thrombectomy are selected for reperfusion therapy. Concurrently, the gynecology manages active or impending AUB, while the internal medicine manages traditional stroke risk factors such as blood pressure, blood glucose, and lipid levels over the long term.

In the remission and long-term management, the gynecology selects conservative treatments or hysterectomy based on fertility preservation preferences. When conservative treatments (GnRH-a, LNG-IUS, curettage, and endometrial destruction) fail and symptoms recur, hysterectomy remains the best option to prevent CI recurrence once symptoms are stabilized. Neurology determines the timing of antithrombotic therapy based on the method of achieving amenorrhea chosen by gynecology, and evaluates the stroke etiology and selects between antiplatelet and anticoagulation treatments. If vascular stenosis is present, neurosurgical intervention should be incorporated in the comprehensive plan.

This study innovatively and systematically integrates the multifactorial interactive mechanisms underlying CI in adenomyosis patients, proposing a multidisciplinary collaborative and staged intervention treatment pathway. The framework provides a scientific foundation and practical approach for the differential diagnosis and precise treatment of these patients, providing valuable clinical reference.

## Author contributions

**Data curation:** Shan Jiang.

**Conceptualization:** Wanting Ji, Zanhui Jia.

**Funding acquisition:** Zanhui Jia.

**Investigation:** Liuqing Yang, Jiaqi Wen.

**Methodology:** Liuqing Yang.

**Project administration:** Zanhui Jia.

**Supervision:** Yuanyuan Cao.

**Validation:** Yuanyuan Cao.

**Writing – original draft:** Shan Jiang.

**Writing – review & editing:** Liuqing Yang.
